# Extranodal natural killer/T-cell lymphoma with tonsil involvement: a case report

**DOI:** 10.1186/s12903-024-04452-x

**Published:** 2024-07-30

**Authors:** Yang Xiao, Xing Zhang, Yingqin Gao, Ken Lin, Wenyue Chi, Kaijian Zhou, Jing Ma, Tiesong Zhang

**Affiliations:** 1 Department of Otolaryngology, Head and Neck Surgery, Children’s Hospital Affiliated to Kunming Medical University (Kunming Children’s Hospital), Kunming, China; 2 Department of Cardiology, Children’s Hospital Affiliated to Kunming Medical University (Kunming Children’s Hospital), Kunming, China; 3 Kunming Medical University Haiyuan College, Kunming, China

**Keywords:** Natural killer/T-cell lymphoma, ENKTL, Tonsils, SMILE chemotherapy, Case report

## Abstract

**Background:**

Extranodal natural killer/T-cell lymphoma (ENKTL) with tonsil involvement is not common, especially in children.

**Case presentation:**

A 13-year-old girl presented with an unexplained sore throat for more than 2 months, together with intermittent fever and suppurative tonsilitis. Nasopharyngoscopy revealed a pharyngeal mass. Enhanced computed tomography (CT) scan showed tonsillar hypertrophy and punctate calcification. Chronic pyogenic granulomatous inflammation with pseudoepithelial squamous epithelial hyperplasia was observed in left tonsil, and pyogenic granulomatous inflammation and a small number of T-lymphoid cells were detected in the right tonsil. The immunohistochemical results showed CD2^+^, CD3^+^, CD4^+^, CD5^+^, CD8^+^, granzyme B^+^, and TIA-1^+^. The Ki-67 proliferation index was 20%. The case showed T cell receptor gene rearrangement. Finally, the case was diagnosed as ENKTL of stage II with tonsil involvement. The patient received 6 cycles of chemotherapy with SMILE regimen, and showed complete response with no recurrence in the follow-up.

**Conclusion:**

We presented a rare case of ENKTL with tonsil involvement in a child. The patient showed complete response to the SMILE chemotherapy with no recurrence.

## Introduction

Natural killer/T-cell lymphoma (NKTL) is an aggressive malignant tumor of NK cell or cytotoxic T cell lineage origin [1]. The disease is more prevalent in Asia, Central and South America than in Western countries [2, 3]. In China, NKTL is one of the most common lymphoma types, second only to diffuse large B-cell lymphoma [4].

Extranodal NKTL (ENKTL), designated as a typical type of NKTL, most often occurs in non-lymphatic sites such as nose [5], nasopharynx [6], and upper aerodigestive tract in adults [7]. Additionally, the skin and gastrointestinal tract may also be involved in patients of an advanced stage [8]. To our best knowledge, few or even no cases with tonsil involvement have been reported in children and adolescents. Here, we present a rare case of ENKTL with tonsil involvement in a child.

## Case presentation

**Fig. 1:**
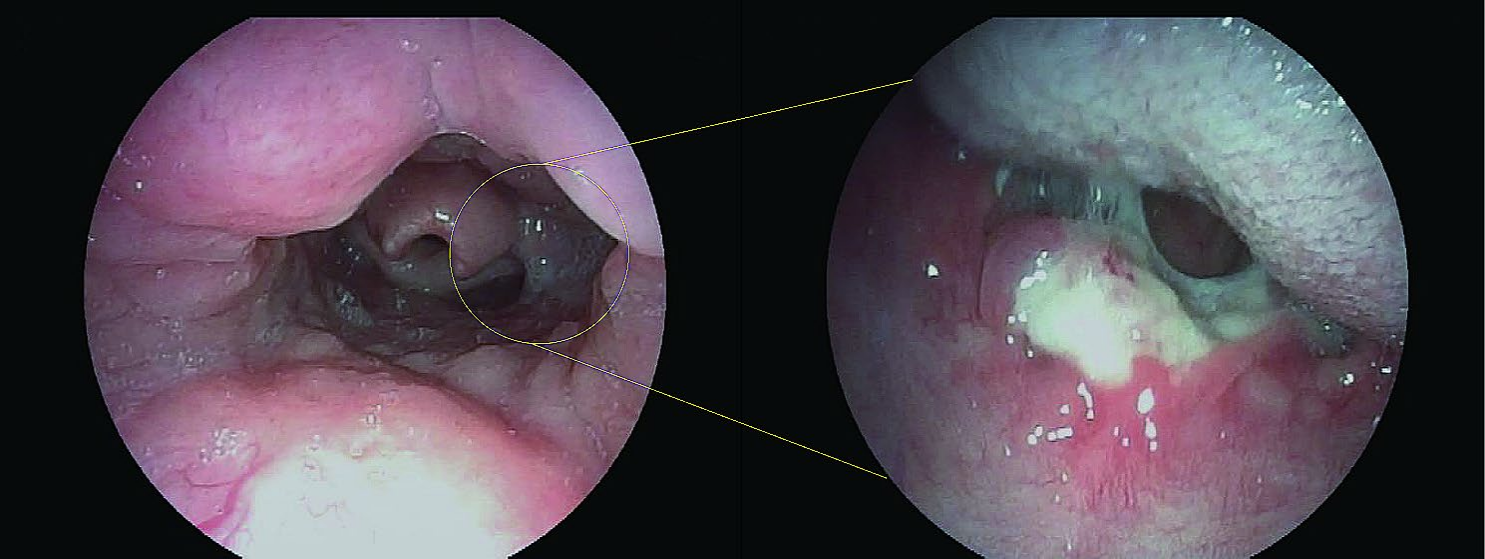
Nasopharyngoscopy indicated an ulcer lesion in pharyngeal region

**Fig. 2:**
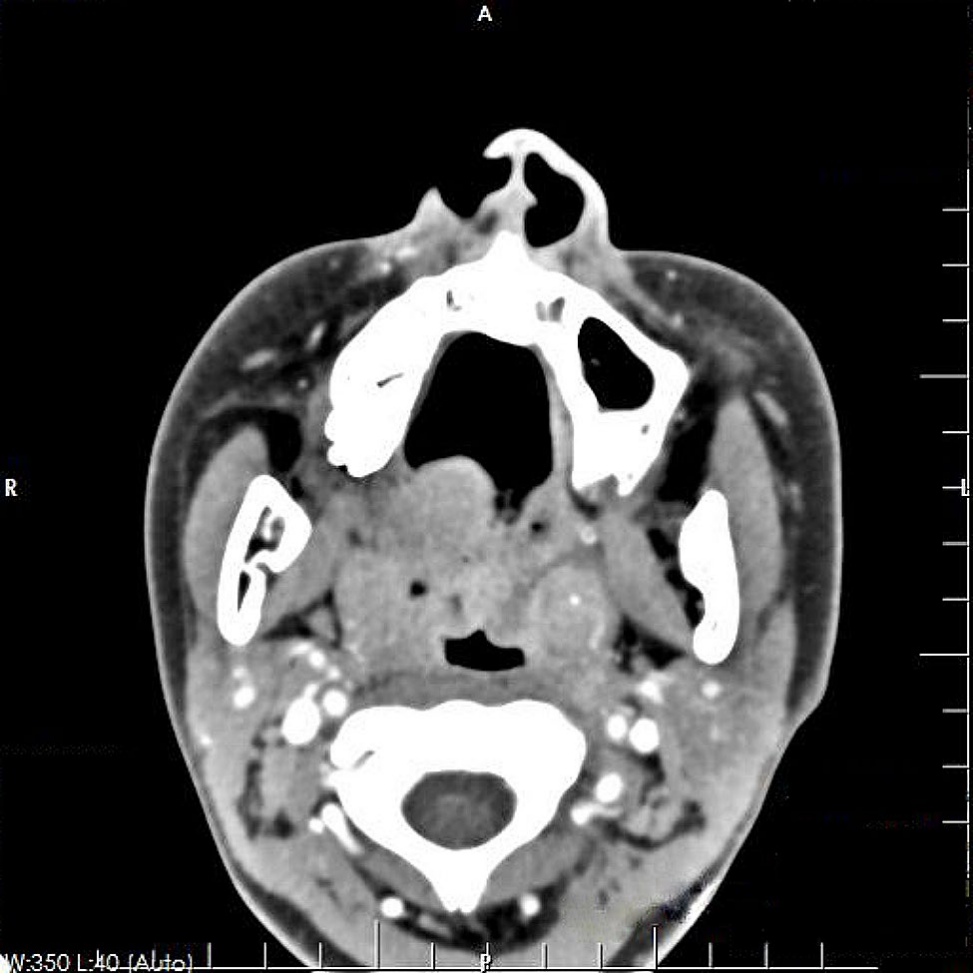
Contrast enhanced CT showed a slightly swollen uvula, bilateral tonsillar enlargement, and punctate calcification of the left tonsil

A 13-year-old girl complained about sore throat for more than 2 months accompanied by intermittent fever. She was diagnosed with suppurative tonsillitis in a local hospital before admitting to our hospital, and denied history of convulsions or coma, nausea, vomiting, or difficulty swallowing. Since the onset of the disease, she showed a poor appetite and poor-quality sleep. Nasopharyngoscopy revealed an ulcer lesion mass in pharyngeal region (Fig. 1). Contrast enhanced computed tomography (CT) showed slightly swollen uvula and bilateral tonsillar enlargement (Fig. 2). In the absence of surgical contraindications, tonsil biopsy was performed under general anesthesia after obtaining the written informed consent from her parents.

**Fig. 3:**
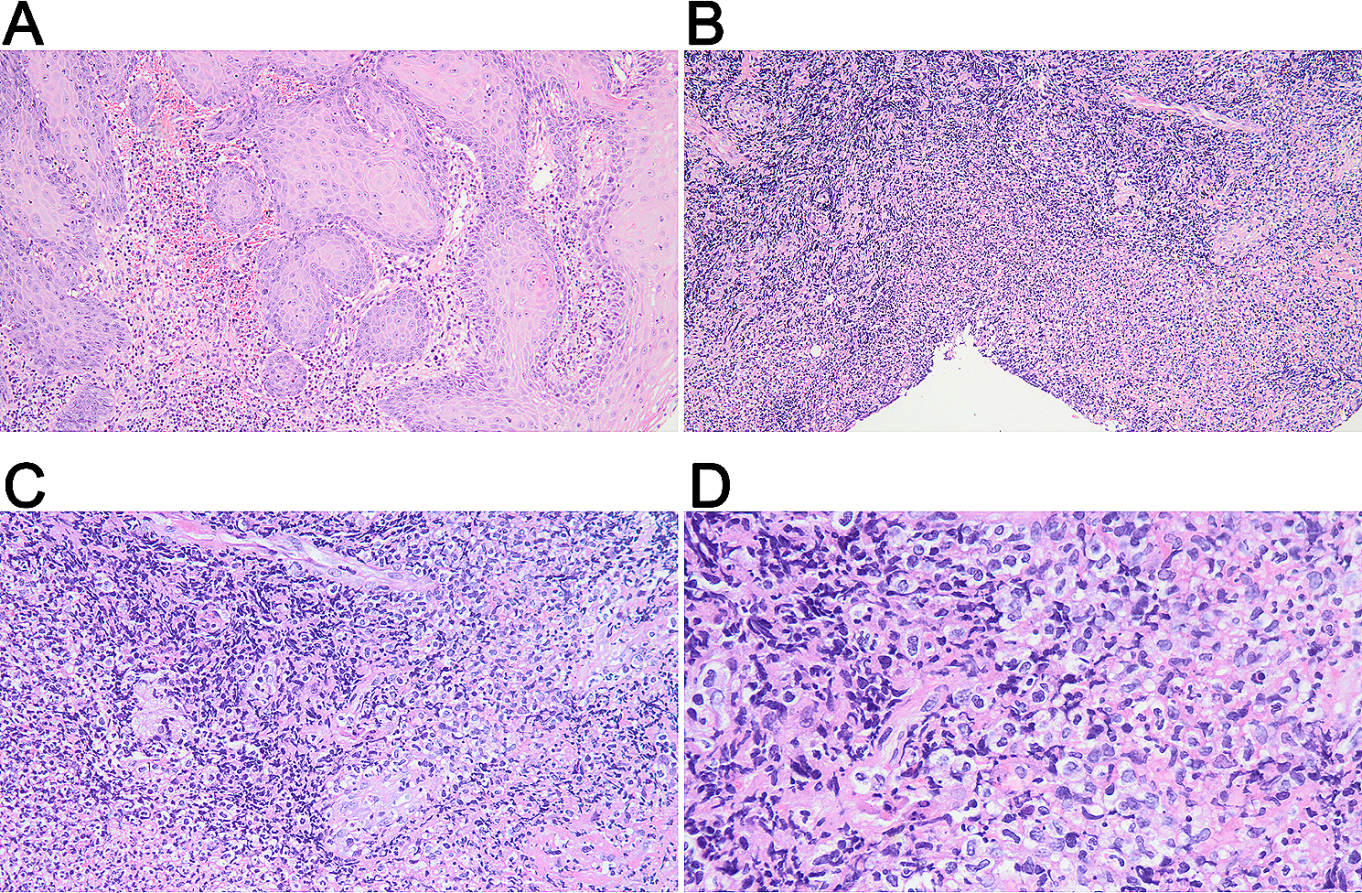
Representative HE-staining images of tonsil mass. **(A)** Perilesional pseudoepitheliomatous hyperplasia, under a magnification of 100×; **(B)** Massive necrosis and vascular proliferation, under a magnification of 100×; **(C)** Small foci of tumor cells distributed in the necrotic background and invading blood vessels, under a magnification of 200×; **(D)** Tumor cells have medium, translucent cytoplasm and irregular, distorted nuclei, under a magnification of 400×

**Fig. 4:**
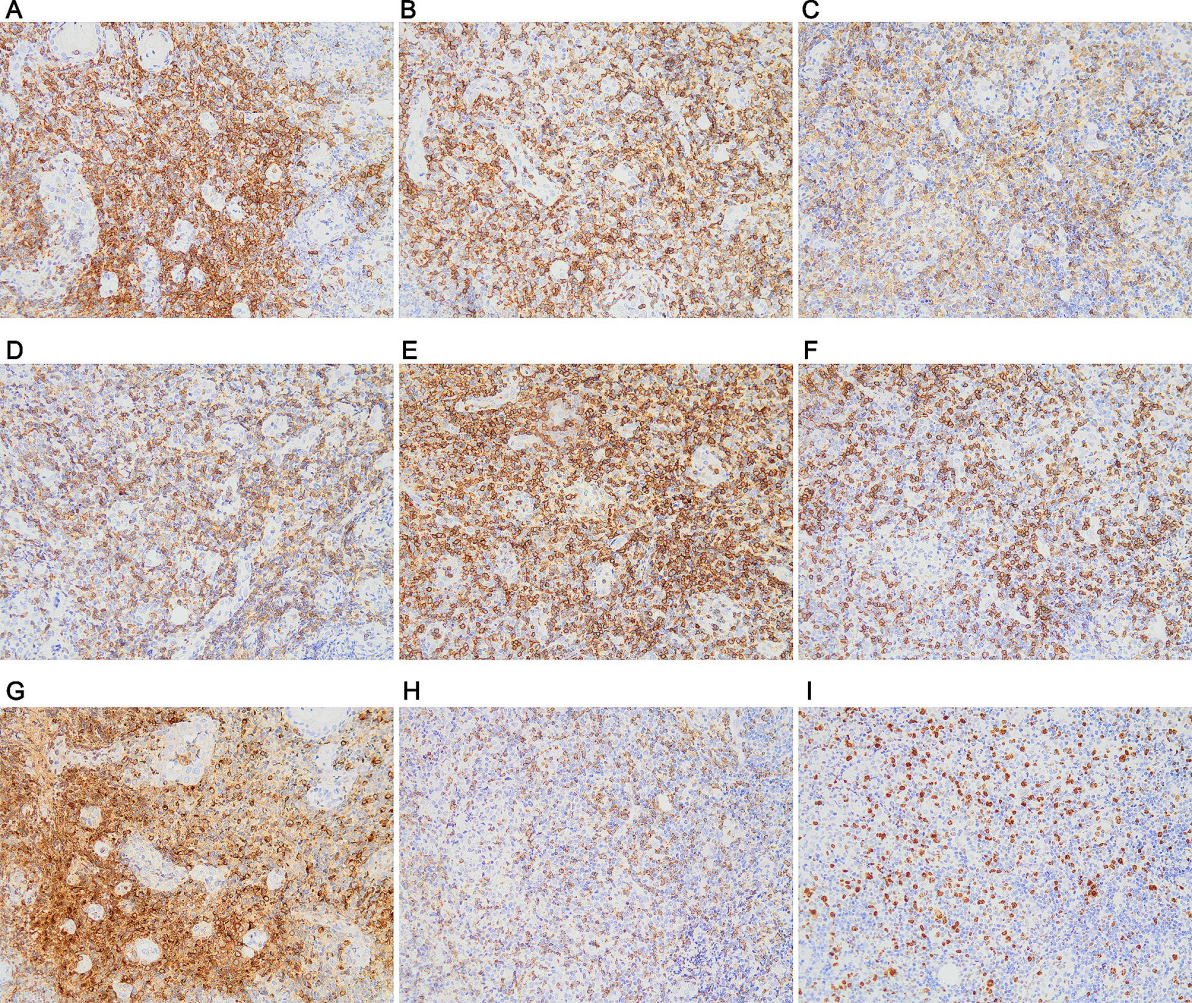
Immunohistochemical results of tonsil mass. The tumor cells were positive for CD2 **(A)**, CD3 **(B)**, CD4 **(C)**, CD5 **(D)**, CD7 **(E)**, CD8 **(F)**, granzyme B **(G)**, and TIA-1 **(H)**. **(I)**. Ki-67 expression. The images were observed under a magnification of 200×

**Fig. 5:**
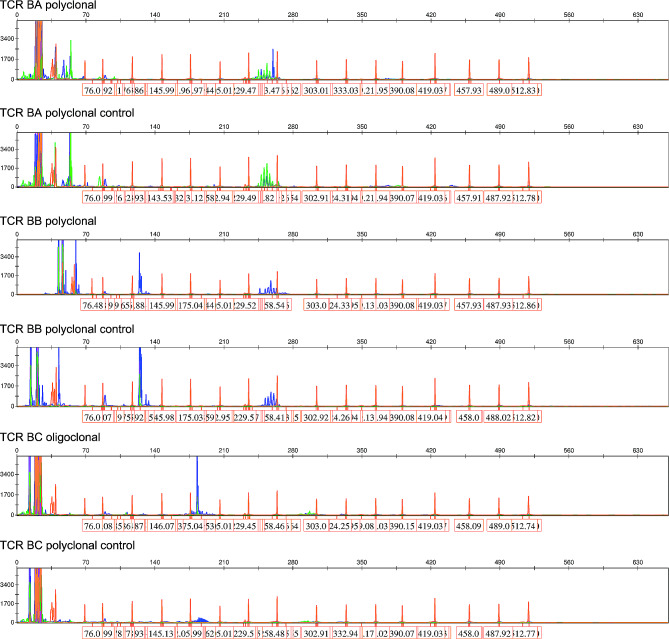
A positive gene scan result of T cell receptor (TCR) gene rearrangement. The clonal rearrangement test utilized PCR-capillary electrophoresis analysis to detect the size distribution of TCR gene amplification. For TCRB-C test, the size range of the PCR amplification product was distributed in the two intervals of 170–210 bp and 285–325 bp. The judgment of monoclonality was based on whether there was a single prominent peak or multiple peaks of similar height within these two ranges. If it was obviously single, it was judged as positive. This TCRB-C obviously had a single blue prominent peak and was judged as positive. For the TCRB-B reaction, the judgment range was 240–285 bp. Within the range, there were multiple blue peaks, and there was no particularly prominent peak. Therefore, it was considered to be negative. NC, negative control

**Fig. 6:**
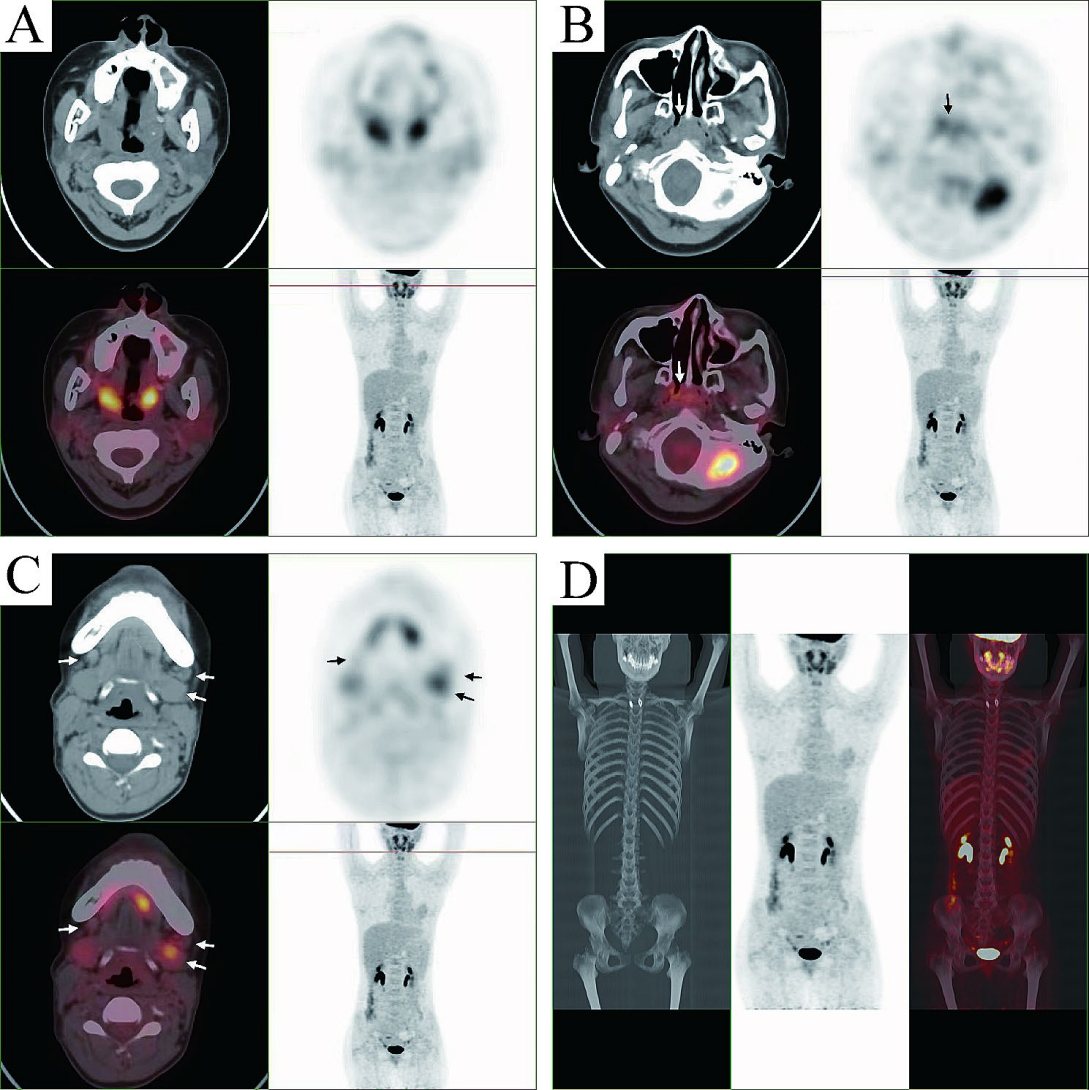
Positron emission tomography/computed tomography (PET/CT) indicated infiltration to the throat **(A)**, maxillary **(B)**, and submandibular glands **(C)**. **(D)** No obvious distant metastasis was detected

Pathological examination indicated tonsillar ulcer and necrosis, underlying blood vessels and lymphocytes proliferation. Chronic pyogenic granulomatous inflammation was observed together with pseudoepithelial squamous epithelial hyperplasia in left tonsil, and pyogenic granulomatous inflammation and a small number of T-lymphoid cells were detected in the right tonsil. Some lymphocytes showed bright cytoplasm, round, oval or slightly irregular nuclei, together with multiple lymphocytes and neutrophils infiltration, as well as presence of a few glands. Lymphocyte infiltration was seen in a few vessel walls. In addition, the tonsillar surface showed stratified squamous epithelial pseudoepithelial hyperplasia, vascular hyperplasia, necrosis, and acute and chronic inflammatory cell infiltration (Fig. 3). Immunohistochemically, the tumor cells were positive for CD2 (Fig. 4A), CD3 (Fig. 4B), CD4 (Fig. 4C), CD5 (Fig. 4D), CD7 (Fig. 4E), CD8 (Fig. 4F), granzyme B (Fig. 4G), and TIA-1 (Fig. 4H), and were negative for CD20, CD57, CD34 and SMA (data not shown). The Ki-67 proliferation index was 20% (Fig. 4I). On this basis, NKTL was highly suspected, and then genetic test was performed. T cell receptor (TCR) gene rearrangement was detected (Fig. 5). PET/CT showed infiltration mainly to the tonsil and posterior pharyngeal wall (Fig. 6) with the maximal SUV of 8.3 and 7.5, respectively. Finally, the patient was diagnosed with stage II ENKTL with tonsil involvement.

For the treatment, the patient received 6 cycles of chemotherapy using SMILE regimen (Table 1**)**. The patient showed complete response with no recurrence or adverse events in the 12-month follow-up.

**Table 1:** SMILE chemotherapy scheme

Agent	Standard dose/day	Actual dose/day	Day
Inpatient medication			
Methotrexate	2 g/m^2^	5.6 g/m^2^	1
Leucovorin	15 mg	SD	2, 3, 4
Ifosfamide	1.5 g/m^2^	1.65 g/m^2^	2, 3, 4
Mesna	300 mg/m^2^	SD	2, 3, 4
Dexamethasone	40 mg	SD	2, 3, 4
Etoposide	100 mg/m^2^	110 mg/m^2^	2, 3, 4
Outpatient medication			
L-asparaginase	6000 U/m^2^	SD	8, 10, 12, 14, 16, 18, 20

The chemotherapy lasted for 28 days. SD, standard dose; SMILE, steroid (dexamethasone), methotrexate, ifosfamide, L-asparaginase, and etoposide

## Discussion

ENKTL is rarely reported in children and adolescents [9], with most cases showing nasopharyngeal involvement. Occasionally, extranasal involvement has been reported, including skin, lungs, intestinal tract, testis, and bone marrow [10]. However, tonsil involvement in children has been rarely reported. We presented a 13-year-old girl of ENKTL with tonsils involvement, and reported our experiences on it with an aim to provide enhanced understanding on it.

Given that ENKTL mostly occurred in the nasopharynx, patients often presented with nasal congestion, epistaxis, fever, and swollen lymph nodes [11]. As its early clinical manifestations are atypical, it is usually misdiagnosed as lymphatic diseases. For the differential diagnosis, ENKTL should be distinguished from indolent T-cell lymphoproliferative disease. Indolent T-cell lymphoma showed low proliferation and no tendency of metastasis. In contrast, ENKTL showed a high possibility of metastasis with fast proliferation speed. Pathologically, T lymphocyte infiltration was the major feature for indolent T-cell lymphoma, while that for the ENKTL usually involved diffused lymphocyte infiltration and lymphoid follicle hyperplasia. At the same time, lymphoma gene rearrangement can also be used to identify these two conditions. In clinical practice, giant lymph node hyperplasia is characterized by lymphatic enlargement with unknown etiology, which mainly invades the chest cavity, hilum and lungs. Chronic lymphadenitis often has obvious lesions, concurrent with localized lymphadenopathy, headache, and tenderness. Such possibility was excluded as the patient showed no related symptoms.

The diagnosis of ENKTL mainly relies on biopsy and immunohistochemical staining, as well as TCR gene rearrangement. The histological phenotypes of ENKTL were often diffuse and permeative lymphocytic proliferation with an angiocentric and angiodestructive growth pattern, as well as fibrinoid changes [12]. ENKTL is typically composed of intermediate-sized cells with focal transformed cells or mixed small and large cells [13], which show irregular nuclear contours, inconspicuous nucleoli, and granular chromatin [14]. Consistent with previous reports, the pathological findings of our case included proliferation in blood vessels and lymphocytes, a portion of bright lymphocyte cytoplasm, profuse infiltration of lymphocytes and neutrophils, as well as round, oval or slightly irregular nuclei. In terms of immunophenotype, the typical phenotype of ENKTL was CD2^+^, cytoplasmic CD3 epsilon^+^, and cytotoxic markers (granzyme B, TIA-1, and perforin) (+) [3, 15]. Consistently, the immunohistochemical results for the tonsillar samples in this study were CD2^+^, CD3^+^, CD4^+^, CD5^+^, CD8^+^, granzyme B^+^, and TIA-1^+^. The expression rates of these markers reported in previous studies conducted using large cohorts were CD2 (93%), cCD3 (84%), CD4 (10%), CD5 (27%), CD8 (22%), granzyme B (83%), TIA1 (90%), and perforin (86%) [16–18]. Ki-67 proliferation index has been reported to be highly correlated with disease progression [19]. The moderate Ki-67 index (20%) in the case indicated moderate progression and moderate risk of the disease. In most cases, TCR genes were in germ-line configuration, but a small proportion of cases may show clonal TCR gene rearrangement that indicated T-cell derivation [20, 21]. Finally, the patient was diagnosed as ENKTL with tonsil involvement.

To date, there is still no standard treatment regimen for ENKTL. As previously described, the most successful treatment option is the non-anthracycline-containing regimens, especially with L-asparaginase [22, 23]. Up to 90% of the patients of stage I and II would show remission after treatment [8]. In a retrospective cohort study included 336 NKTL patients, cases received asparaginase-containing chemotherapy showed higher overall response rates and complete remission rates than the counterparts received no asparaginase-containing regimens. Consistently, our case underwent asparaginase-based SMILE chemotherapy showed good outcome with complete response.

## Conclusion

We reported a 13-year-old ENKTL case with tonsil involvement based on CT, pathological analysis and TCR gene rearrangement. The patient showed complete response after SMILE chemotherapy. We hope that this case will be of interest to readers and helpful to the diagnosis, treatment, risk assessment, and management of ENKTL.

## Data Availability

All data generated or analysed during this study are included in this published article.

## Abbreviations

CT: computed tomography
ENKTL: extranodal natural killer/T-cell lymphoma
NKTL: natural killer/T-cell lymphoma
TCR: T cell receptor
